# Characterization of the Estradiol-Binding Site Structure of Human Protein Disulfide Isomerase (PDI)

**DOI:** 10.1371/journal.pone.0027185

**Published:** 2011-11-03

**Authors:** Xin-Miao Fu, Pan Wang, Bao Ting Zhu

**Affiliations:** 1 Department of Pharmacology, Toxicology and Therapeutics, School of Medicine, University of Kansas Medical Center, Kansas City, Kansas, United States of America; 2 State Key Laboratory of Protein and Plant Gene Research, School of Life Sciences, and the Center for Protein Science, Peking University, Beijing, China; University of Kent, United Kingdom

## Abstract

**Background:**

Earlier studies showed that 17β-estradiol (E_2_), an endogenous female sex hormone, can bind to human protein disulfide isomerase (PDI), a protein folding catalyst for disulfide bond formation and rearrangement. This binding interaction can modulate the intracellular levels of E_2_ and its biological actions. However, the structure of PDI's E_2_-binding site is still unclear at present, which is the focus of this study.

**Methodology/Principal Findings:**

The E_2_-binding site structure of human PDI was studied by using various biochemical approaches coupled with radiometric receptor-binding assays, site-directed mutagenesis, and molecular computational modeling. Analysis of various PDI protein fragments showed that the [^3^H]E_2_-binding activity is not associated with the single *b* or *b'* domain but is associated with the *b-b'* domain combination. Computational docking analyses predicted that the E_2_-binding site is located in a hydrophobic pocket composed mainly of the *b'* domain and partially of the *b* domain. A hydrogen bond, formed between the 3-hydroxyl group of E_2_ and His256 of PDI is critical for the binding interaction. This binding model was jointly confirmed by a series of detailed experiments, including site-directed mutagenesis of the His256 residue coupled with selective modifications of the ligand structures to alter the binding interaction.

**Conclusions/Significance:**

The results of this study elucidated the structural basis for the PDI–E_2_ binding interaction and the reservoir role of PDI in modulating the intracellular E_2_ levels. The identified PDI E_2_-binding site is quite different from its known peptide binding sites. Given that PDI is a potential therapeutic target for cancer chemotherapy and HIV prevention and that E_2_ can inhibit PDI activity *in vitro*, the E_2_-binding site structure of human PDI determined here offers structural insights which may aid in the rational design of novel PDI inhibitors.

## Introduction

Protein disulfide isomerase (PDI) is a 57-kDa oxidoreductase of the thioredoxin superfamily that is expressed mainly in the endoplasmic reticulum (ER) of eukaryotic cells. This protein plays a vital role in the folding of many proteins in the ER by serving as a catalyst of disulfide rearrangements (isomerase activity), disulfide formation (oxidase activity), and disulfide reduction (reductase activity) [1]–[5]. In addition, studies have shown that PDI is also involved in many other important biological processes, such as viral infection (*e.g.*, HIV-1 fusogenic events), through its reduction of the disulfide bonds in toxin and related factors [6], [7], ER stress response [8], [9], and others.

Besides serving as a protein folding catalyst, a number of studies have shown that PDI can also serve as an intracellular binding protein for certain small molecules that contain a phenolic structure, which includes both endogenous hormones (estrogens [10] and thyroid hormones [11], [12]) and environmental chemicals (*e.g.*, endocrine disruptors) [13]. It has been suggested [12] that PDI, owing to its unusually high levels present in various types of cells in rodents as well as humans, may function as an effective intracellular reservoir for estrogenic hormones and thus may modulate their intracellular availability as well as total content in target cells. This concept was supported by our recent study showing that PDI can modulate the intracellular levels of 17β-estradiol (E_2_) in human breast cancer cells, augment its hormonal activity, and slow down its metabolic disposition [14]. Given that E_2_ is an important female sex hormone and plays vital physiological roles in the human body [15], [16] and that PDI is ubiquitously expressed in various cells and tissues [17], [18], it is expected that PDI may serve as a global modulator of the intracellular levels and actions of E_2_ in humans. On the other hand, the observed ability of E_2_ and particularly some of its structural analogs to interact with human PDI may also have pharmacological relevance given that some of these compounds can effectively inhibit PDI's catalytic activity *in vitro* [10], [12], [13], [19] and that PDI has received considerable attention in recent years as a potential therapeutic target in cancer chemotherapy [20], [21] and HIV prevention [22]–[25].

At present, the E_2_-binding site structure of human PDI is not known. The main purpose of our present study, therefore, was to delineate the structural basis of human PDI's E_2_-binding activity. Through a combined use of various biochemical approaches coupled with radiometric ligand-receptor binding assays, computational modeling, site-directed mutagenesis, and selective ligand modifications, we located the PDI's E_2_-binding site to a hydrophobic pocket between the *b* and *b'* domains. In addition, we have built the PDI-E_2_ binding model, and identified a critical hydrogen bond formed between PDI-His256 and the 3-hydroxyl group of E_2_. This is the first characterization of the E_2_-binding site structure of human PDI. The findings offer mechanistic insights at the molecular level concerning the structural basis of the PDI–E_2_ binding interaction and its reservoir role in modulating the intracellular E_2_ levels.

## Results

### Biochemical characterization of the E_2_-binding site in human PDI protein

**Figure 1:**
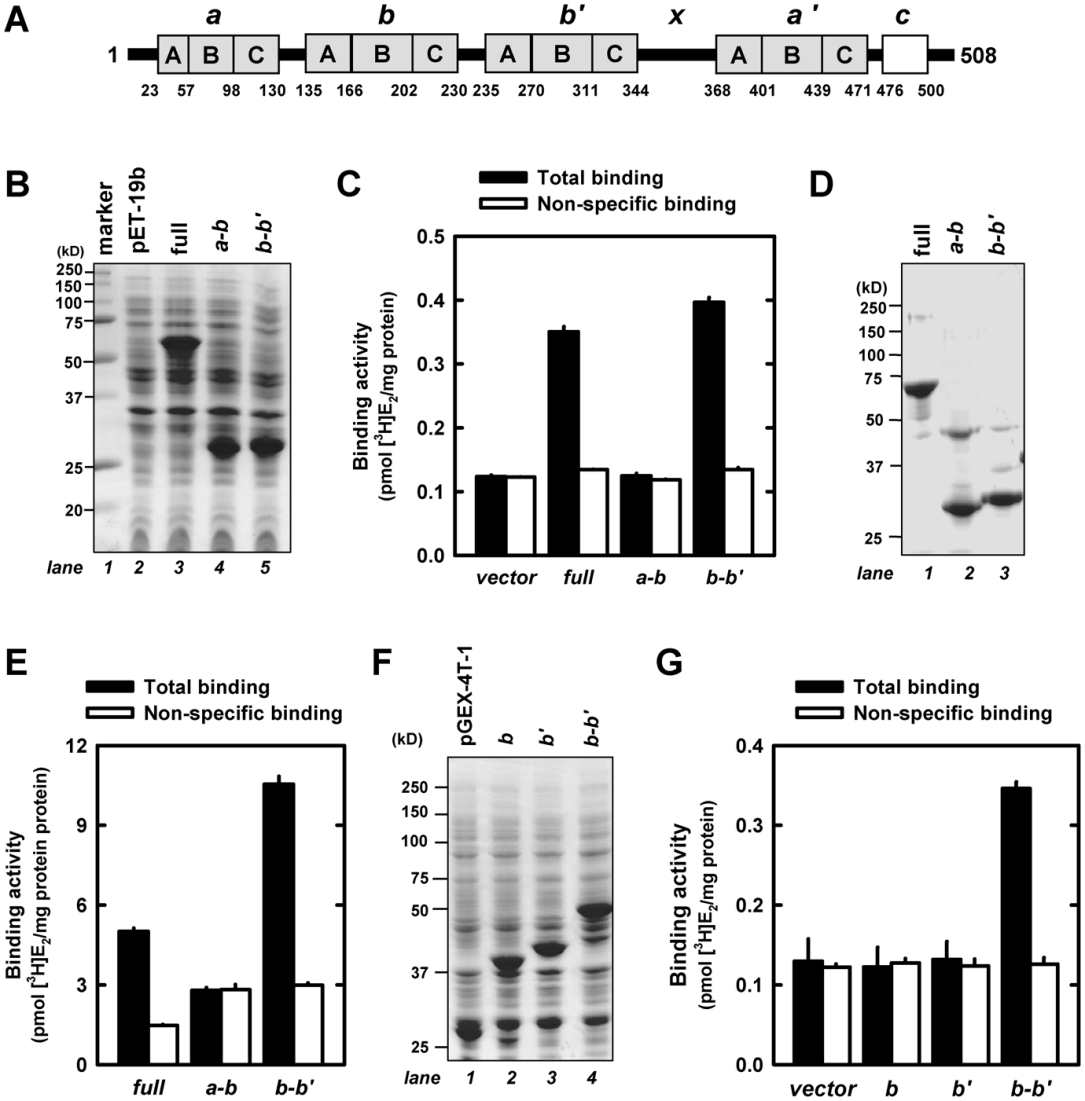
Both PDI and its *b-b'* fragment can bind E_2_. (**A**). Domain organization of the human PDI protein. The letters A, B and C in boxes that represent the β-α-β, α-β-α, and β-β-α secondary-structure elements, respectively, of the thioredoxin fold, are adopted from an earlier study [40] and were used to guide the design of various PDI protein fragments as shown in this figure and Figure S1. (**B**) and (**D**). SDS-PAGE analysis of two histidine-tagged PDI fragments and the full-length protein, which were selectively expressed in *E. coli* cells (panel **B**) and then purified using affinity chromatography (panel **D**). (**C**) and (**E**). The binding of [^3^H]E_2_ by either cell lysates (at a final protein concentration of 1 mg/mL; panel **C**) or by purified proteins (at a final concentration of 0.5 µM; panel **E**) after incubation with 4.5 nM [^3^H]E_2_ in the absence or presence of 10 µM non-radioactive E_2_. (**F**). SDS-PAGE analysis of selectively-expressed GST-tagged PDI protein fragments in *E. coli* cells. (**G**). The binding of [^3^H]E_2_ by cell lysates containing the GST-tagged PDI fragments. For the quantitative data, each value is the mean ± S.D. of triplicate determinations.

Recently, we have characterized the E_2_-binding site structure of human PDIp [26], which shares similar domain architecture with human PDI [27]. Both PDI and PDIp are multi-domain proteins composed of four thioredoxin-like domains, *a*, *b*, *b'* and *a'*, plus a small linker region *x* between *b'* and *a'* and a *C*-terminal acidic extension *c* (as depicted in Figure 1A). We hypothesized that human PDI may have a similar E_2_-binding site structure as that of human PDIp, which binds E_2_ in a hydrophobic pocket between its *b* and *b'* domains [28]. To test this hypothesis, we first designed two truncated human PDI fragments (namely, *a-b* and *b-b'*), with a histidine tag attached to their *N*-termini for the convenience of purification, and then their [^3^H]E_2_-binding activities were determined and compared with that of the full-length human PDI protein. After these two PDI fragments were cloned and selectively expressed in *E. coli* cells (left part in Figure 1B), they were purified (Figure 1D). Radiometric [^3^H]E_2_-binding assay using whole cell lysates (Figure 1C) and purified proteins (Figure 1E) both showed that the *b-b'* fragment can bind E_2_ as does the full-length protein. However, no binding activity was detected for the *a-b* fragment when it was assayed under the same conditions. In addition, we have also prepared several other PDI fragments that contain the *a'* domain, including *b'-x-a', x-a'-c, b'(C)-x-a'-c,* and *b'(BC)-x-a'-c* (for structures of these fragments, see Figure 1A), to test their potential [^3^H]E_2_-binding activity. As shown in Figure S1C, none of them was found to have any appreciable [^3^H]E_2_-binding activity. Together, these results clearly suggest that the E_2_-binding site is only associated with the *b*-*b'* fragment.

Next, we selectively expressed the single *b* and *b'* domains for testing their individual E_2_-binding activity. In our initial experiments, we also adopted the same strategy of attaching a histidine tag to the single domain fragment. However, we did not observe any E_2_-binding activity for these histidine-tagged single domain proteins (Figure S1C). To confirm this negative result, we also prepared the GST-tagged *b* and *b'* fusion proteins, in which GST, as a structurally more stable protein, might help these small protein fragments fold better and thus better exert their potential E_2_-binding activity. The cell lysates containing the GST-tagged *b* or *b'* domain (Figure 1F, lane 2, 3) also did not exhibit any appreciable [^3^H]E_2_-binding activity (Figure 1G). The failure of the GST fusion proteins to bind [^3^H]E_2_ is not due to the potential interference of the GST tag because the GST-tagged *b-b'* fragment (Figure 1F, lane 4) retains strong [^3^H]E_2_-binding activity (Figure 1G). Collectively, these observations suggest that the intact E_2_-binding site of PDI is not associated with the single *b* or *b'* domain but is associated with the natural *b* and *b'* domain combination (*i.e.*, the *b-b'* fragment).

**Figure 2:**
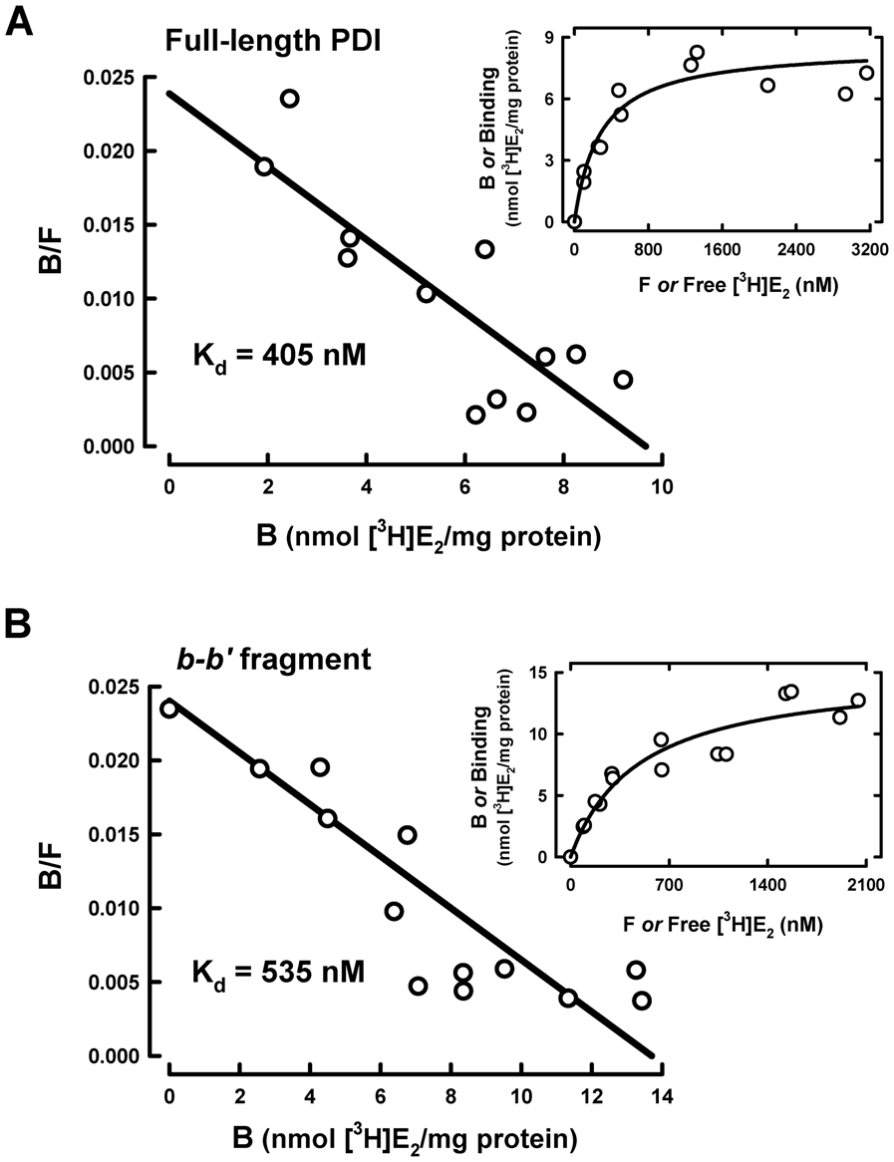
Determination of the dissociation constant (K_d_) of the full-length human PDI protein (A) and its *b-b'* fragment (B) for E_2_. Equilibrium analysis was used to determine the dissociation constants as described in the Materials and Methods section. The concentrations of free and total [^3^H]E_2_ (*i.e.*, the sum of PDI-bound [^3^H]E_2_ and free [^3^H]E_2_) were determined by scintillation counting calibrated against standard concentrations of [^3^H]E_2_ (see Figure S2B). Total [^3^H]E_2_ subtracted by free [^3^H]E_2_ gives rise to PDI-bound [^3^H]E_2_. The upper right insets show the binding curves were obtained using curve regression analysis (hyperbola model) of the SigmaPlot software. Each value is the mean of duplicate determinations.

To further probe the intactness of the E_2_-binding site in the *b-b'* fragment, we compared its E_2_-binding affinity (*i.e.*, the K_d_ value) with the full-length PDI protein. The *in vitro* equilibrium dialysis assay showed that the apparent K_d_ values for [^3^H]E_2_ binding (calculated according to their Scatchard plots) are 405±82 nM for the full-length protein (Figure 2A) and 535±86 nM for the *b-b'* fragment (Figure 2B). The difference in the estimated K_d_ values of the full-length human PDI protein and its *b-b'* fragment for [^3^H]E_2_ binding is not statistically significant. Based on their Scatchard plots, it is apparent that both PDI and its *b-b'* fragment exhibited single binding site kinetics, suggesting that each of them only has a single E_2_-binding site. Here it is also of note that the binding affinity of PDI for E_2_ as determined in this study is higher than that reported earlier [12], likely due, in part, to the different incubation temperatures used in these binding assays, and/or to different PDI species used (human PDI in this study and rat PDI in the earlier study).

### Computational docking analysis of the E_2_-binding site structure of PDI *b-b'* fragment

Because the E_2_-binding site is only associated with the *b-b'* fragment and also because the *b-b'* fragment has the same E_2_-binding affinity as that of the full-length PDI, we thus used the known PDI *b-b'* fragment structure [28] for computational docking analysis to probe the E_2_-binding site structure. First, we used the *Insight II* modeling program to search for the cavities present in the protein structure. Four cavities were found in the *b-b'* fragment (Figure S3): cavity I is located between the *b* and *b'* domains, *i.e.*, it is formed jointly by these two domains (cavity size  =  300 Å^3^). In comparison, cavity IV is solely formed by the *b* domain, and cavity II and cavity III are solely formed by the *b'* domain. Since the location of cavities II, III and IV in the single *b'* or *b* domain is not supported by the biochemical results (shown in Figure 1 and S1), they were not further studied. Instead, cavity I was the only cavity that was selected for computational docking analysis of its binding interaction with E_2_.

**Figure 3:**
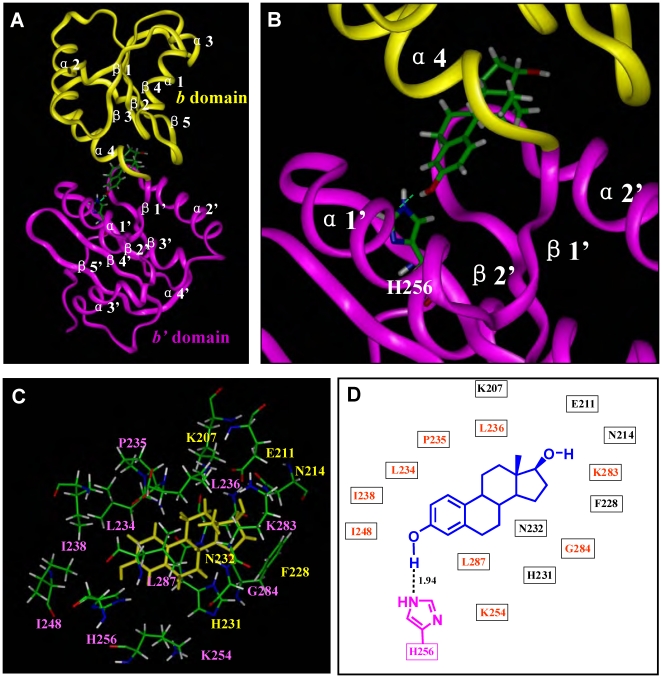
Docking analysis of the binding interaction of E_2_ inside human PDI *b-b'* fragment. (**A**). Overview of the docking result of the E_2_ binding in the PDI *b-b'* fragment. E_2_ and His256 are shown in the ball-and-stick format and colored according to atoms. The protein structure is shown in ribbon. Yellow colored region denotes the *b* domain and magenta colored region denotes the *b'* domain. Secondary structural elements are labeled according the NMR structure of the fragment. (**B**). A close-up view of the docking result of the E_2_-PDI binding mode, showing that a hydrogen bond (by green dash) is formed between the 3-hydroxyl group of E_2_ (a hydrogen bond donor) and PDI-His256 (a hydrogen bond acceptor). (**C**). Interaction of E_2_ with the amino acid residues inside the binding pocket. Labeling of amino acid residues is shown in yellow for the *b* domain and in magenta for the *b'* domain. E_2_ molecule is colored in yellow. Amino acid residues are shown in the ball-and-stick format and colored according to atoms, *i.e.*, green for carbon, red for oxygen, white for hydrogen, and blue for nitrogen. (**D**). Plots of the docking result of the E_2_ binding with PDI *b-b'* fragment. The distance is in angstroms. E_2_ is colored in blue and His256 is in magenta. Note that the amino acid residues of the *b* and *b'* domains are colored differently (black for the *b* domain and red for the *b'* domain).

The overall E_2_-PDI binding interaction as revealed by docking analysis is shown in Figure 3A and 3B, and the amino acid residues surrounding the binding pocket are shown in Figure 3C. A few features can be readily drawn from this binding model (Figure 3B, 3C, 3D). First, one hydrogen bond is formed between the 3-hydroxyl group of E_2_ and the nitrogen atom of PDI-His256, whereas no hydrogen bond is formed between the 17-hydroxyl group of E_2_ and PDI. In this respect, the E_2_-PDI interaction is quite similar to the E_2_-PDIp interaction [26]. Second, the major binding site is contributed by the hydrophobic residues, which provide strong hydrophobic interactions with the four aliphatic rings of E_2_. Most of the hydrophobic residues are from the *b'* domain (*i.e.,* F228, L234, L236, I238, L287, and I248), while only one is from the *b* domain (F228). Third, the polar residues surrounding the binding site are mainly from the *b'* domain (*i.e.*, H231, N232, P235, K254, H256, K283, and G284), but the *b* domain (*i.e.*, K207, E211, and N214) also contributes, to certain degrees. Together, the computational docking results show that the *b'* domain provides the principal E_2_-binding site whereas the *b* domain plays a relatively minor role.

### Role of the hydrogen bond between 3-hydroxyl group of E_2_ and His256

**Figure 4:**
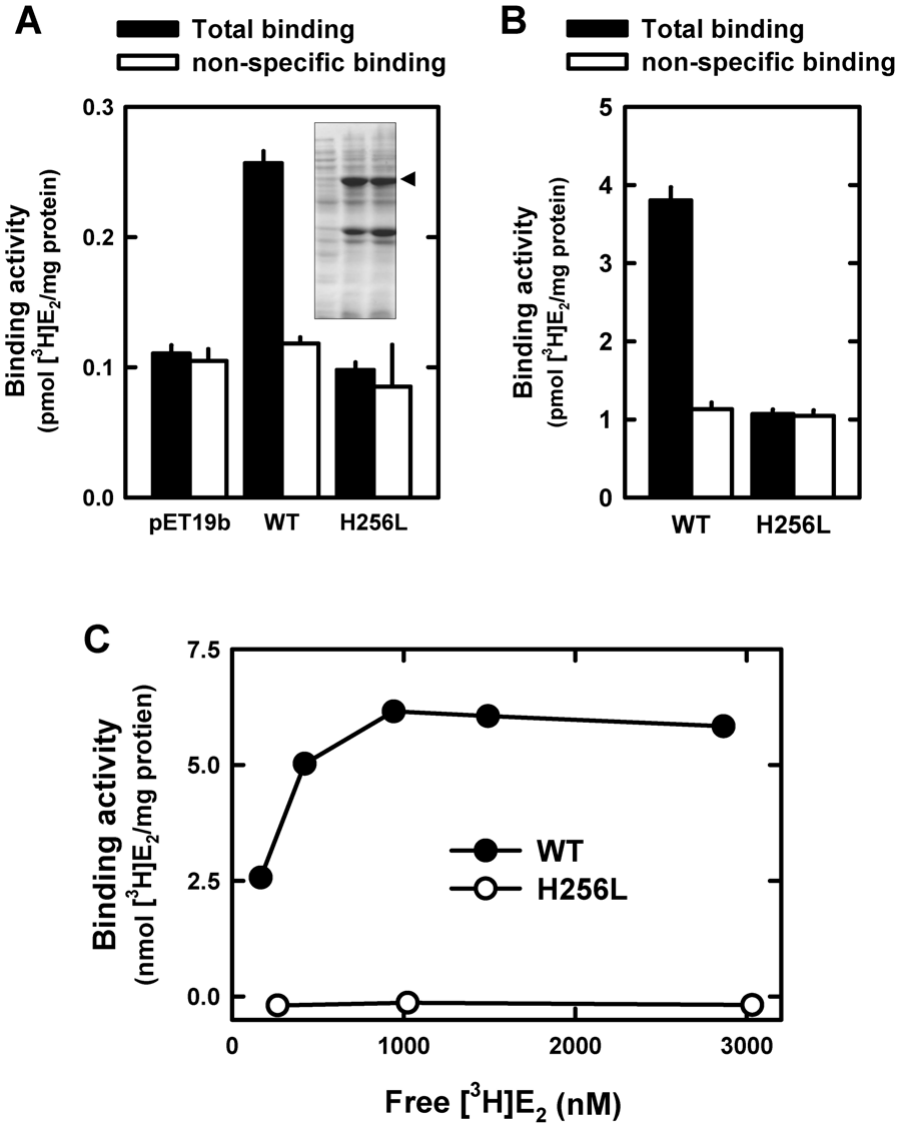
H256L mutant protein lacks E_2_-binding activity. (**A**) and (**B**). The [^3^H]E_2_ binding by *E. coli* cell lysates containing the selectively-expressed wild-type or H256L mutant DPI proteins (panel **A**) or by purified proteins (panel **B**) after incubation with 4.5 nM [^3^H]E_2_ in the absence or presence of non-radioactive E_2_ in excess (10 µM). Inset in panel **A** shows the SDS-PAGE analysis of the cell lysates. (**C**). The PDI-bound [^3^H]E_2_ (wild-type and H256L mutant proteins) against increasing concentrations of [^3^H]E_2_ (200 to 3000 nM). Equilibrium analysis was used to determine the binding activity as described in the Materials and Methods section. The concentrations of free and total [^3^H]E_2_ (*i.e.*, the sum of PDI-bound [^3^H]E_2_ and free [^3^H]E_2_) were determined by scintillation counting calibrated against standard concentrations of [^3^H]E_2_ (see **Figure S2B**).

To experimentally test the binding model as suggested by our computational docking analysis, we first sought to determine whether the identified amino acid residue, His256, is indeed involved in the binding interactions between PDI and E_2_ through forming a hydrogen bond. According to earlier studies [29], [30], the side-chain length of leucine is similar to that of histidine but the former does not contain electro-negative atoms that are necessary for the formation of hydrogen bonds. Therefore, we mutated His256 to leucine (H256L). As shown in Figure 4A and 4B, neither the whole cell lysates that contain the full-length H256L mutant PDI protein nor the purified mutant protein retain any appreciable [^3^H]E_2_-binding activity when compared with the wild-type PDI protein. The radiometric ligand-receptor binding assay further confirmed that this mutant protein does not have an appreciable [^3^H]E_2_-binding activity even when very high concentrations of [^3^H]E_2_ are present (Figure 4C). Similarly, we also created the H256L mutation in the *b-b'* fragment and found that this mutant fragment does not have any [^3^H]E_2_-binding activity (*data not shown*). Taken together, these data clearly show that the His256 residue of human PDI is indispensable for its binding interaction with E_2_.

**Figure 5:**
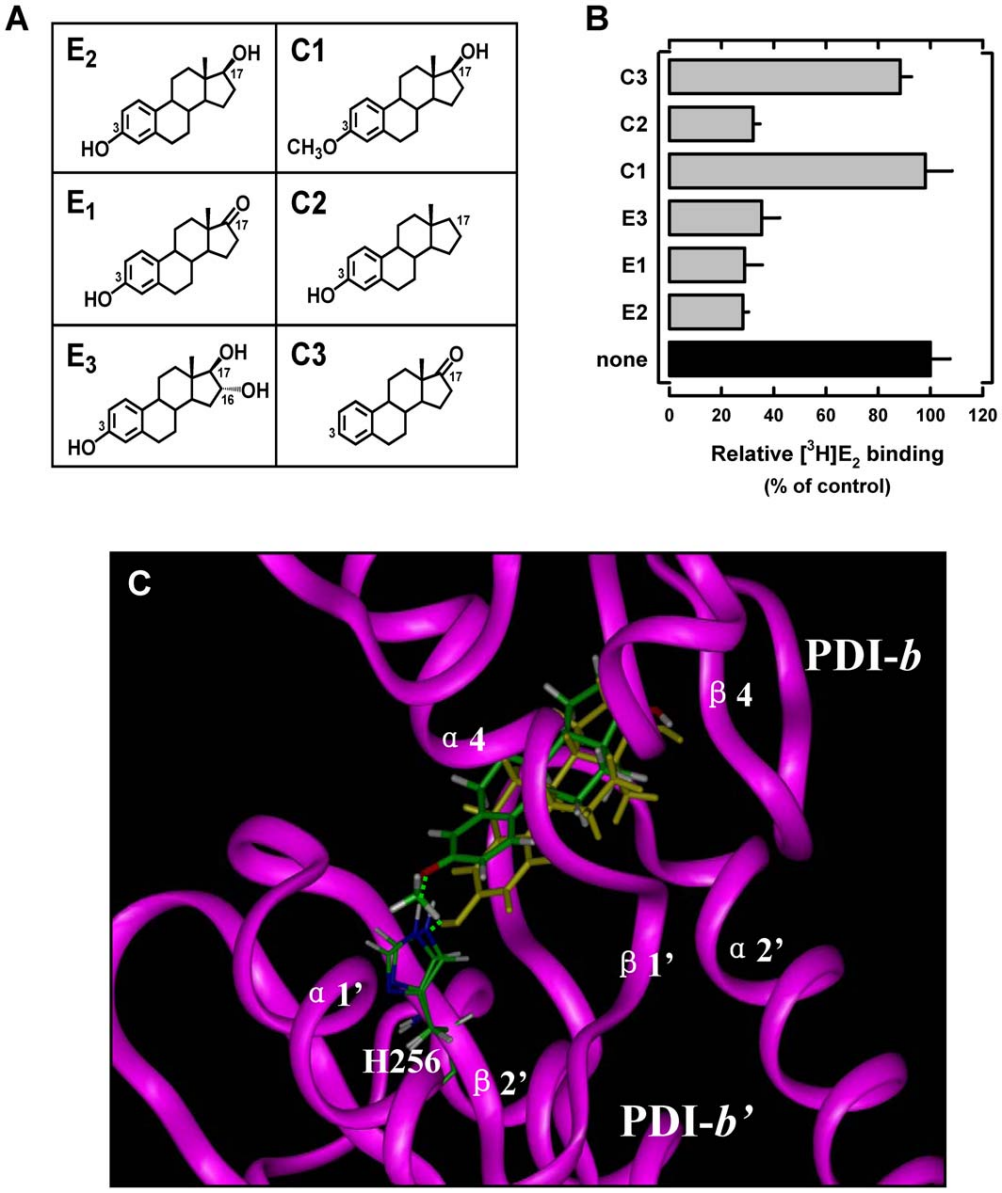
Relative binding activity of PDI for several E_2_ derivatives. (**A**). Chemical structures of E_2_ and several of its analogs used in this study. (**B**). Relative binding activity of the purified PDI protein (at 20 µg/mL final concentration, purified from *E. coli* cells) for [^3^H]E_2_ (4.5 nM) in the absence or presence of non-radioactive 10 µM E_2_ or its analogs in sodium phosphate buffer (10 mM, pH 7.4). (**C**). Docking analysis of the binding modes of E_2_ and C1 in the binding pocket of the PDI *b-b’* fragment. Protein structure is shown in ribbon and colored in magenta. E_2_, C1 and His278 are shown in the ball-and-stick format. C1 and His 256 are colored according to atoms and E_2_ colored yellow. Green dashes denote hydrogen bonds. α-Helics and β-sheets are labeled according to Figure S3.

To provide further experimental support for the conclusion derived from the mutagenesis experiments, we employed an alternative approach by using a number of E_2_ derivatives that share the same core structure as E_2_ but with their C-3 or C-17 hydroxyl group selectively modified such that they cannot form the same type of hydrogen bonds with human PDI as does E_2_. In these experiments, the purified full-length human PDI protein was used as a binding protein to evaluate the ability of these structurally-modified E_2_ derivatives to compete off the binding of [^3^H]E_2_. We found that E_1_, E_3_, and C2, all of which contain an intact 3-hydroxyl group but differ in their 17-position substitutions (structures shown in Figure 5A), can efficiently compete with [^3^H]E_2_ for binding to the full-length PDI (Figure 5B). This observation is consistent with the computational modeling data, which predicted that the 17-hydroxyl group of E_2_ is not important for its binding interaction with human PDI. In contrast, C3, which lacks the 3-hydroxyl group (Figure 5A), does not have any appreciable activity to compete with [^3^H]E_2_ for PDI binding (Figure 5B). Again, this result is also fully consistent with the computational model, *i.e.*, the 3-hydroxyl group of E_2_ is crucial for PDI-E_2_ binding interaction through forming a hydrogen bond with PDI-His256.

Lastly, it is of note that in the binding model as shown in Figure 3, the 3-hydroxyl group of E_2_ is a hydrogen bond donor rather than an acceptor, while the nitrogen atom of PDI-His256 is a hydrogen bond acceptor rather than a donor. Evidence in support of this computational prediction came from the experimental testing of the relative binding activity of 3-methoxyestradiol (structure shown in Figure 5A as C1) for human PDI. Theoretically, the oxygen atom in the 3-methoxyl group of this E_2_ derivative can only serve as a hydrogen bond acceptor but not a hydrogen bond donor in forming hydrogen bonds. As expected, computational docking analysis showed that 3-methoxyestradiol can still bind comfortably inside the binding pocket in nearly the same manner as does E_2_, suggesting that its larger 3-methoxyl group does not cause steric hindrance of its binding (Figure 5C). In addition, the computational model also suggests that 3-methoxyestradiol might form a hydrogen bond with His256, but in this case, the hydrogen bond would be very different from the hydrogen bond formed with E_2_, *i.e.*, the 3-methoxyestradiol serves as a hydrogen bond acceptor while PDI-His256 serves as a hydrogen bond donor (Figure 5C). However, experimental testing of its binding activity for human PDI revealed that 3-methoxyestradiol does not retain any appreciable binding activity (Figure 5B). This observation suggests that the potential hydrogen bond as predicted by the computational model likely is very weak (of negligible significance). More importantly, the experimental observation provides additional support for the predicted binding model as shown in Figure 3, *i.e.*, the hydrogen bond is formed in a highly specific orientation between E_2_ and His256, with the former serving as a hydrogen bond donor and the latter as a hydrogen bond acceptor.

## Discussion

**Figure 6:**
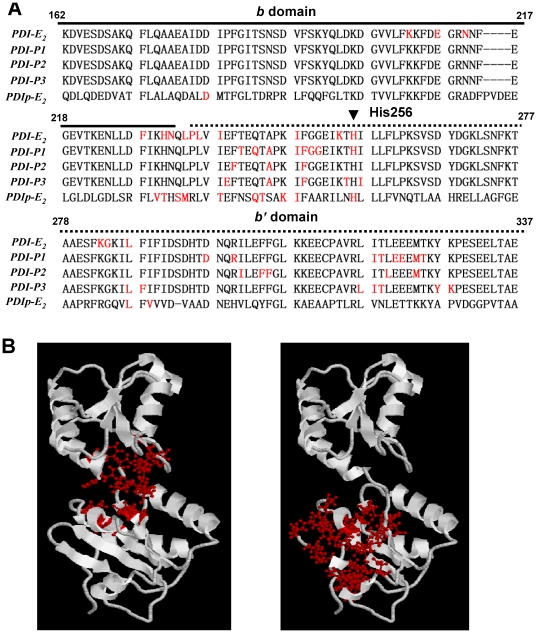
Amino acid sequence alignment showing the overlap and difference between PDI's E_2_-binding site and its peptide-binding sites. (**A**) Sequence alignment was performed between human PDI and PPIp using Clustal W. The E_2_-binding sites of human PDI and PDIp, which are colored in red, are based on the results of this study and our earlier study [26], respectively, along with the structural information for the peptide-binding sites of PDI determined earlier by others (*i.e.*, the NMR titration analysis of PDI-P1 [28] and PDI-P2 [32], and the structure analysis of PDI-P3 [33]). The arrow indicates those highly-conserved amino acid residues, namely, His256 in PDI and His278 in PDIp, which are essential for their binding interaction with E_2_. Based on an earlier study [28], the essential amino acid residue His256 in PDI is also involved in binding mastoparan (a peptide substrate) and unfolded RNase A [28]. (**B**). Mapping the E_2_-binding site (left part) and peptide-binding site (right part) in the three-dimensional structure of PDI *b-b'* fragment. Peptide-binding sites include the combined residues from PDI-P1, PDI-P2, and PDI-P3 as shown in panel **A**. Binding sites (red) are shown in the ball-and-stick format and the whole protein (white) in a cartoon format.

We recently have characterized the E_2_-binding site structure of human PDIp [26]. Overall, the human PDI and PDIp proteins share similar E_2_-binding site structures, which are located in the hydrophobic pocket composed mainly of the *b'* domain and partially of the *b* domain. Sequence alignment also shows (Figure 6A) that overall the E_2_-binding sites of PDI and PDIp are quite conserved and nearly located in the same region. The 3-hydroxyl group of E_2_ is crucial for its binding interaction with both PDI (Figures 3,4,5,6) and PDIp [26], through forming a hydrogen bond with PDI-His256 and PDIp-His278, respectively. These two histidine amino acids are conserved in these two proteins (as indicated by the arrow in Figure 6A), but are not found in Erp57 and PDIA6, two other PDI family members that were recently found to have no E_2_-binding activity [31]. In addition, the hydrophobic amino acid residues inside the E_2_-binding pockets of both PDI (Figure 3) and PDIp [26] may also contribute to the E_2_ binding through hydrophobic interactions. A contributing role of the hydrophobic interactions in PDI-E_2_ binding is partly supported by our experimental observation showing that Triton X-100 (at low concentrations of <0.02%, *v/v*), a commonly used hydrophobic detergent, can effectively inhibit the E_2_-binding activity of PDI and PDIp (data not shown).

Earlier studies using the NMR titration [28], [32] and structure analysis [33] have determined the peptide-binding site of PDI, which was found to be located in a solvent-exposed hydrophobic pocket [33]. The amino acid sequence alignment analysis of the E_2_-binding site of PDI and its peptide-binding site (Figure 6A) shows that there is only a limited degree of overlap between these two binding sites. Mapping of these two sites in the three-dimensional structure of PDI *b-b'* fragment clearly shows that they are very different with one another, suggesting that PDI likely binds peptides and E_2_ in rather different fashions. Nevertheless, the key amino acid residue for E_2_-binding, *i.e.*, His256, appears to be also involved in binding mastoparan (a peptide substrate) and unfolded RNase A [28]. However, whether His256 forms a hydrogen bond with the peptide substrate during its binding interaction remains unknown at present.

It is known that the 3-hydroxyl group of E_2_ plays an essential role in its binding interaction with human estrogen receptors α and β (ERα and ERβ) by forming hydrogen bonds [34]–[36]. The structural model developed in the present study offers a reasonable explanation for the experimental observations that the human PDI has a much lower E_2_-binding affinity than human ERα and ERβ. First, human PDI forms only one hydrogen bond with the 3-hydroxyl group of E_2_, whereas human ERα or ERβ each can form two hydrogen bonds with the 3-hydroxyl group. Second, while the 17β-hydroxyl group of E_2_ plays a negligible role in its binding interaction with human PDI, this hydroxyl group plays an important role in its interaction with human ERα or ERβ [34]. Third, for both ERα and ERβ, almost all amino acid residues in their binding pockets, except those that form hydrogen bonds with E_2_, are hydrophobic residues, which provide stronger hydrophobic interactions with the four aliphatic rings of E_2_. However, in the case of PDI, we noted that several polar residues (*e.g.*, P235, K254, H231, N232, K283, N214, E211, and K207; shown in Figure 3C, 3D) are also present in the binding pocket, and it is speculated that these polar residues may weaken the overall hydrophobic interactions with E_2_.

Our results offer structural insights into the reservoir role of PDI in modulating the intracellular levels and actions of E_2_ in mammalian cells [12], [14]. Although PDI has a much lower binding affinity than human ERα and ERβ [12], [14], it is believed that this intracellular protein still can store up considerable amount of E_2_ inside a cell owing to its unusually high levels present (as high as 100 µM, as estimated in an earlier study [12]). Our recent study showed that the PDI-bound E_2_ molecules are protected from metabolic disposition and can be further released to augment ERα-mediated transcriptional activity in human breast cancer cells [14]. Given that E_2_ has vital physiological functions in humans [15], [16] and that PDI is ubiquitously expressed in various cells and tissues [17], [18], it is speculated that PDI may function as an important global modulator of the intracellular E_2_ levels and actions in various target cells. Additionally, since PDI can also bind thyroid hormones [11], [12] as well as other bioactive compounds such as endocrine disruptors [13], [19], it is possible that this protein may also modulate their biological actions in a similar manner. This possibility merits further investigation.

Lastly, it is of note that in recent years, PDI has received considerable attention as a potential therapeutic target in cancer chemotherapy [20], [21] and HIV prevention [22]–[25]. The clinical usefulness of some of the currently-available PDI inhibitors (such as bacitracin), however, is limited due to the occurrence of nephrotoxicity [37] as well as other adverse effects [38]. Although several newer inhibitors, such as DTNB, pCMBS, PAO, aPAO, and GSAO, have also been made in recent years, they have not been used for therapeutic purposes, partly also due to the existence of non-specific activities and adverse effects [7], [25]. The E_2_-binding site structure of human PDI as determined in our present study provides detailed structural information concerning the binding interactions of small non-peptide molecules with human PDI, and this knowledge is expected to aid in the rational design of new PDI inhibitors in the future. For instance, given that E_2_ is an effective inhibitor of PDI activity *in vitro* [10], [12], [13], [19], it is possible to design novel E_2_-based structural analogs that lack estrogenic activity but have a higher PDI inhibition potency through rationally introducing additional hydrogen bonds, salt bridges, and/or hydrophobic interactions with the amino acid residues in the E_2_-binding pocket.

## Materials and Methods

### Chemicals, reagents, cell lines, and tissues

E_2_ and its structural analogs were purchased from Steraloids (Newport, RI). [^3^H]E_2_ (specific activity of 110 Ci/mmol) was obtained from Perkin Elmer (Waltham, MA). All other chemicals and reagents used in this study were of analytical grade or higher.

### Plasmid construction and protein purification

Human PDI (its cDNA clone was obtained from ATCC, catalog no. 6706839) was cloned into the pET-19b vector at the sites of 5′-NdeI/BamHI-3′. The plasmids for the expression of histidine-tagged PDI protein fragments (*e.g.,* various *a-b, b-b'*, *b*, and *b'* fragments) were constructed by cloning the corresponding cDNA sequences into a modified pET-19b vector as described in our earlier study [31]. Purification of the recombinant histidine-tagged proteins expressed in *E. coli* was carried out as described earlier [31], [39]. The GST-tagged PDI *b, b'* and *b-b'* fragments (fusion proteins) were cloned into a modified pGEX-4T-1 vector containing the NdeI sites at 5′-NdeI/SalI-3′ and expressed in *E. coli* and purified using the same chromatographic method [36], [37]. Site-directed mutagenesis was performed using the Phusion® Site-Directed Mutagenesis Kit obtained from the New England Biolabs (Ipswich, MA, USA) according to the instructions of the manufacturers.

### Radiometric [^3^H]E_2_-binding assay for cell lysates and purified PDI proteins and fragments

For most of the *in vitro* radiometric binding assays, we employed the desalting method to separate the free [^3^H]E_2_ and protein-bound [^3^H]E_2_ as described in our recent study [26]. The full-length human PDI protein and its various fragments were individually incubated with [^3^H]E_2_ (typically at a 4.5-nM final concentration) in 10 mM sodium phosphate buffer (pH 7.4) at 4°C overnight and then subjected to desalting using the PD miniTrap G-25 columns obtained from GE health. Eluted fractions from 0.5 to 1.15 mL were collected for radioactivity measurement.

However, for determining the dissociation constants (K_d_ values) of the purified human PDI protein and its *b-b'* fragment for [^3^H]E_2_, we used the equilibrium dialysis method. The experiments were performed at 4°C using a single-sample DISPO equilibrium dialyzer with a 5-kD molecular mass cut-off membrane obtained from Harvard Apparatus (Holliston, MA, USA; catalog no. of 742201). The purified protein or fragment at appropriately diluted concentrations was placed in one chamber of the dialyzer and varying concentrations of [^3^H]E_2_ were placed in a neighboring chamber. The incubation lasted for 48 h, a sufficiently-long time to ensure that the binding reached equilibrium, which was determined in pilot experiments by following the binding equilibrium time course (representative data are shown in Figure S2).

### Computational analysis of the E_2_ binding site and molecular docking

The E_2_-binding site(s) in the human PDI *b-b'* domain complex (PDB code: 2k18) [28] was determined using the *Active-Site-Search* function in the *Binding-Site* module of *Insight II*. The *site-open-size* parameter was set at 5 Å and the *site-cut-off-size* parameter was set at 150 Å^3^. We defined the binding pocket with amino acid residues within 5 Å reach around the candidate binding site. *Simulated Annealing* docking method in the *Affinity* module was used to dock E_2_ into the candidate binding pocket. Water molecules were excluded and side chains in the binding site were allowed to move freely in the docking analysis. One hundred docking modes were calculated and the ones with the lowest binding energy were chosen for further energy minimizations.

## Supporting Information

Figure S1 **The single b and b' domains lack [^3^H]E_2_-binding activity.** (A). SDS-PAGE analysis of *E. coli* cell lysates containing over-expressed GST-tagged PDI fragments. The *b*'(BC)-*x*-*a*'-*c* and *b*'(C)-*x*-*a*'-*c* fragments represent the fragment b'-x-a'-c lacking the secondary structural element *A* and *AB* of the b' domain, respectively. Secondary structural elements are defined as in the legend of Figure 1A. (B). SDS-PAGE analysis of *E. coli* cell lysates containing selectively-expressed histidine-tagged *b* and *b*' domains. (C). The binding of [^3^H]E_2_ by whole cell lysates (at a final concentration of 1 mg/ml in total proteins) after incubation with 4.5 nM [^3^H]E_2_ in the absence or presence of 10 µM non-radioactive E_2_. Cell lysates containing the GST protein (left part) or the full-length PDI protein was used a positive control. Each value is the mean ± S.D. of triplicate determinations.
(TIF)

Figure S2 **Determination of optimal time and [^3^H]E_2_ concentrations in equilibrium analysis.** (A). Time-dependent equilibrium of [^3^H]E_2_ molecules in diffusing across the semi-permeable membrane in the single-sample DISPO equilibrium dialyzer at 4°C or at room temperature. Incubation at 4°C for 48 h appeared to be sufficient to reach the diffusion equilibrium. (B). Correlation between the radioactivity of [^3^H]E_2_ and concentrations of [^3^H]E_2_ when performing the equilibrium analysis as shown in Figure 2. The calibration curve was used to determine the concentrations of free and total [^3^H]E_2_ concentrations.
(TIF)

Figure S3 **Determination of the potential cavities in PDI for E_2_-binding.** (**A**). Four cavities are identified in PDI *b-b'* fragment by using the *Active-Site-Search* function in the *Binding-Site* module of *Insight II*. Cavity I (yellow), II (gray), III (green), and IV (brown) are colored differently in this figure for ease of recognition. α-Helics and β-sheets are labeled according to their NMR structures. (**B**). The structure shown in this panel is the same structure as shown in panel **A**, but with a 90° horizontal rotation.
(TIF)
