# Supplementary figures and images for: Characterization of the Estradiol-Binding Site Structure of Human Protein Disulfide Isomerase (PDI)
Source: PLoS One. 2011 Nov 3;6(11):e27185. doi: 10.1371/journal.pone.0027185 (PMC3207843; doi:10.1371/journal.pone.0027185)

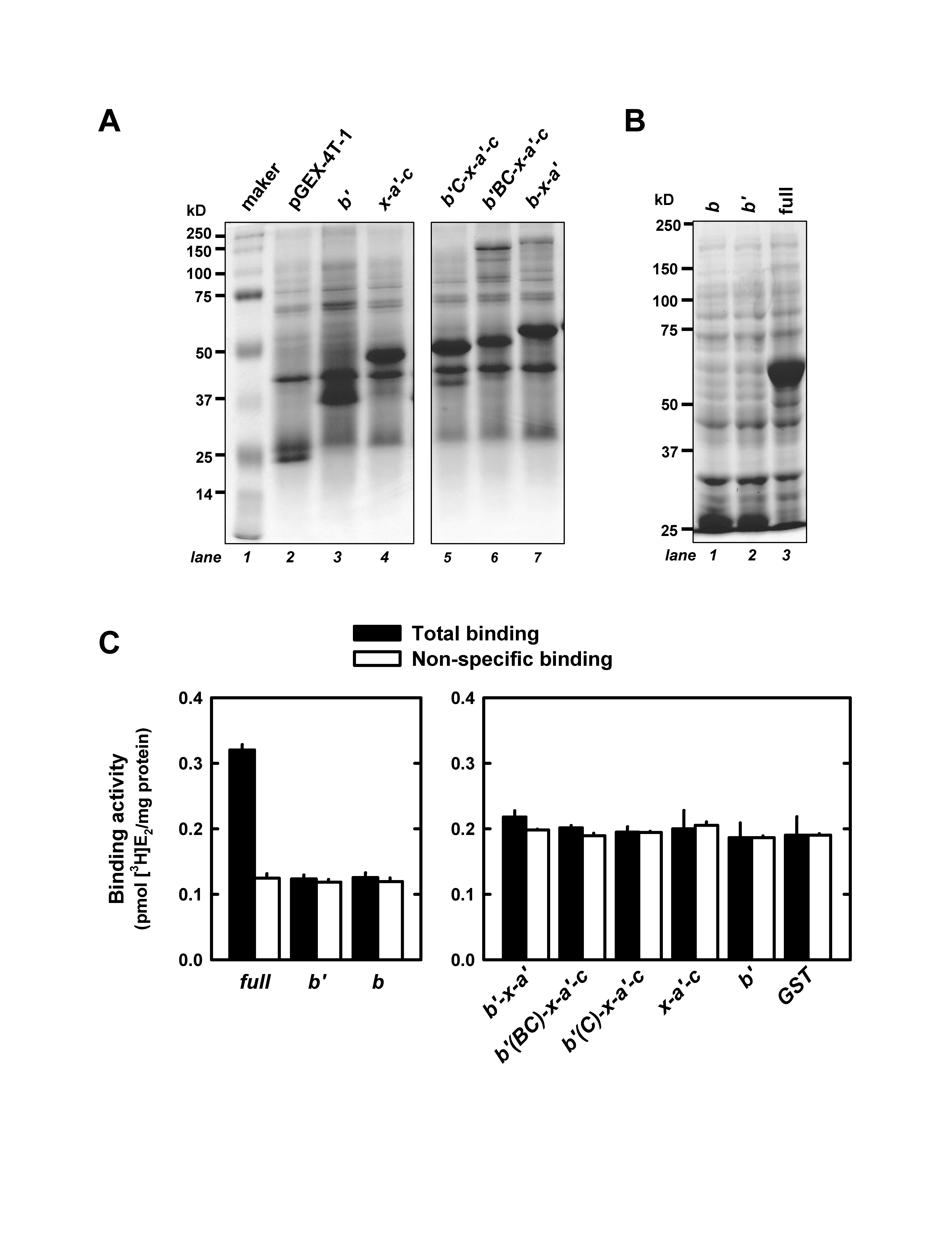

Supplement: Figure S1 — The single b and b' domains lack [3H]E2-binding activity. (A). SDS-PAGE analysis of E. coli cell lysates containing over-expressed GST-tagged PDI fragments. The b'(BC)-x-a'-c and b'(C)-x-a'-c fragments represent the fragment b'-x-a'-c lacking the secondary structural element A and AB of the b' domain, respectively. Secondary structural elements are defined as in the legend of Figure 1A. (B). SDS-PAGE analysis of E. coli cell lysates containing selectively-expressed histidine-tagged b and b' domains. (C). The binding of [3H]E2 by whole cell lysates (at a final concentration of 1 mg/ml in total proteins) after incubation with 4.5 nM [3H]E2 in the absence or presence of 10 µM non-radioactive E2. Cell lysates containing the GST protein (left part) or the full-length PDI protein was used a positive control. Each value is the mean ± S.D. of triplicate determinations. (TIF) [file pone.0027185.s001.tif]

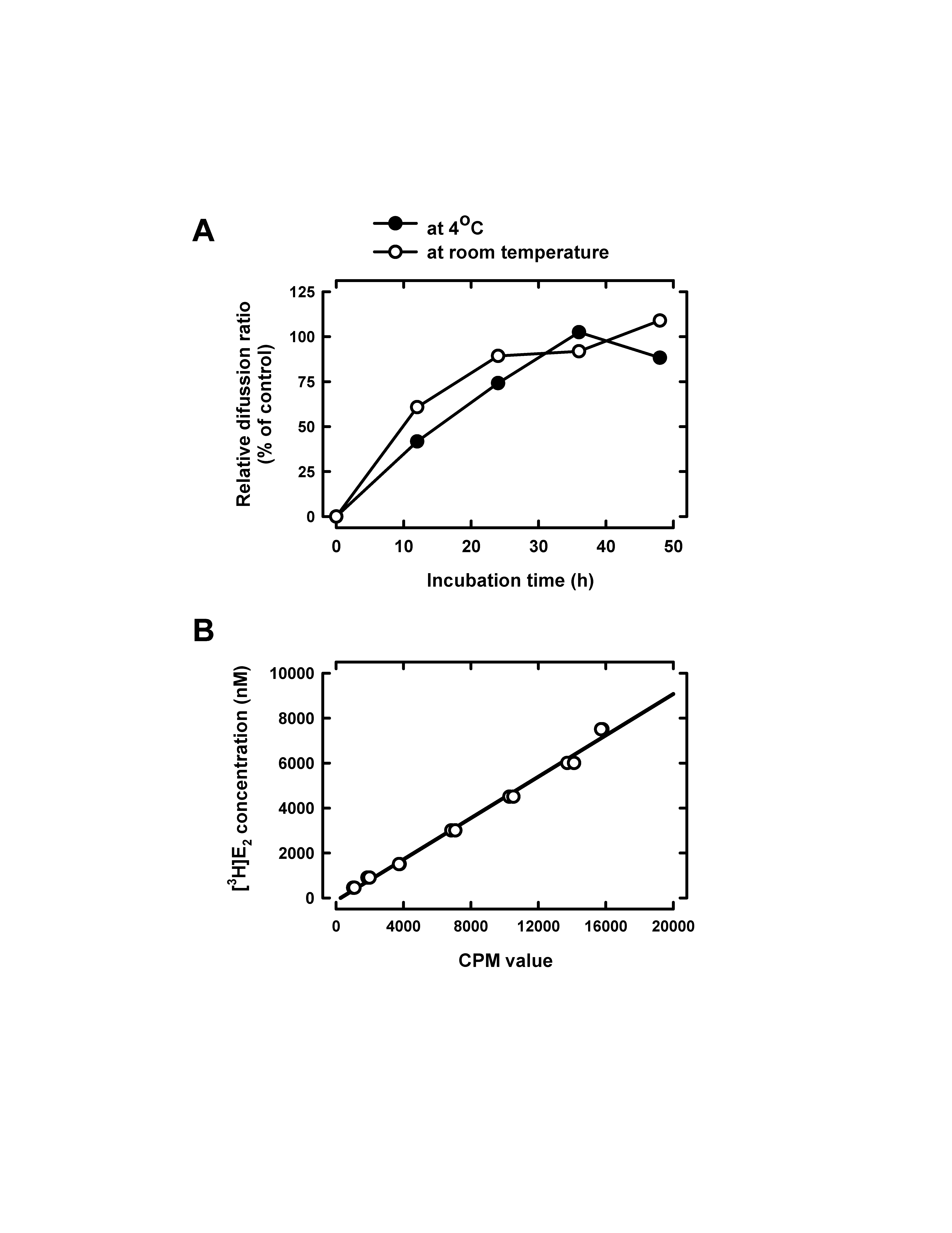

Supplement: Figure S2 — Determination of optimal time and [3H]E2 concentrations in equilibrium analysis. (A). Time-dependent equilibrium of [3H]E2 molecules in diffusing across the semi-permeable membrane in the single-sample DISPO equilibrium dialyzer at 4°C or at room temperature. Incubation at 4°C for 48 h appeared to be sufficient to reach the diffusion equilibrium. (B). Correlation between the radioactivity of [3H]E2 and concentrations of [3H]E2 when performing the equilibrium analysis as shown in Figure 2. The calibration curve was used to determine the concentrations of free and total [3H]E2 concentrations. (TIF) [file pone.0027185.s002.tif]

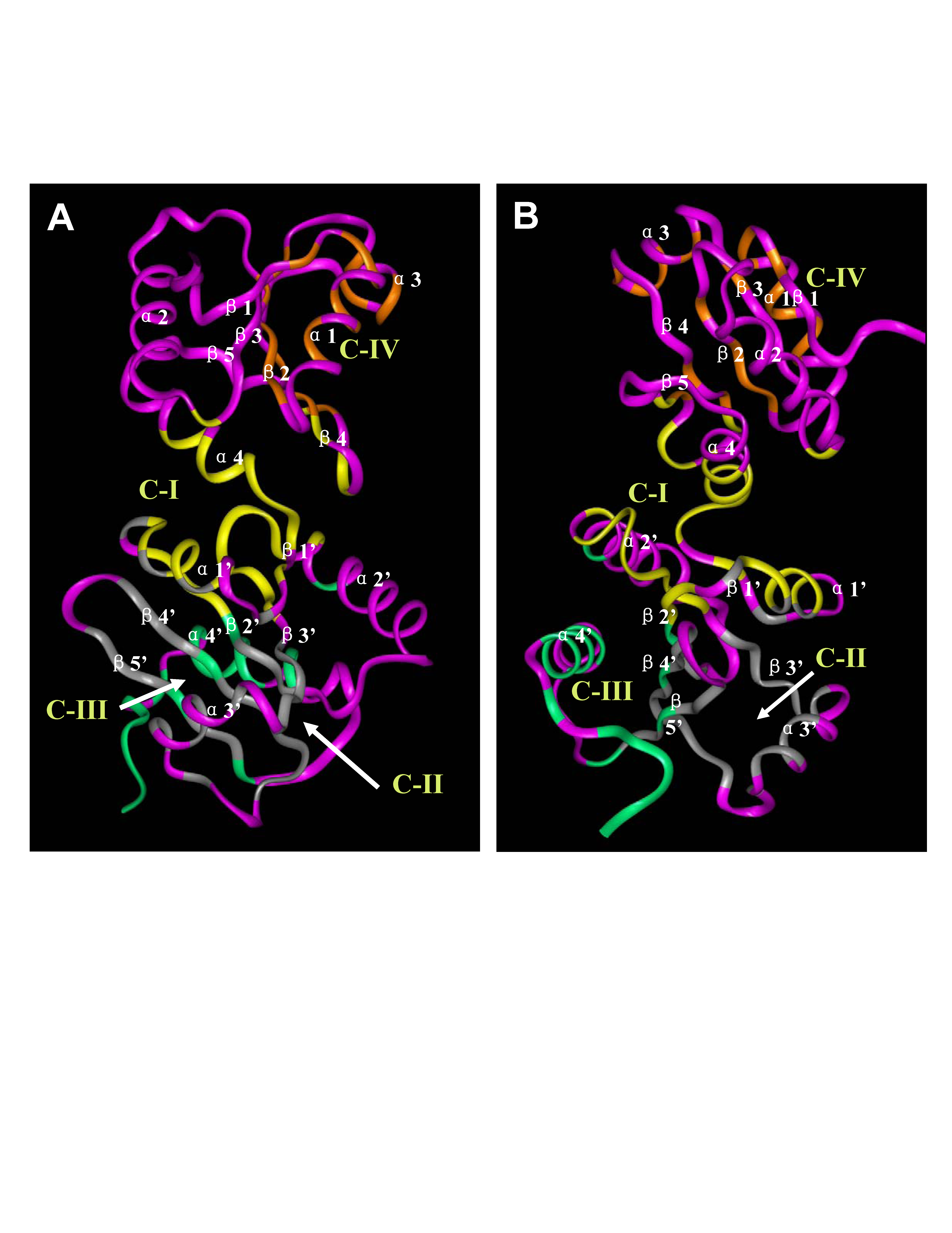

Supplement: Figure S3 — Determination of the potential cavities in PDI for E2-binding. (A). Four cavities are identified in PDI b-b' fragment by using the Active-Site-Search function in the Binding-Site module of Insight II. Cavity I (yellow), II (gray), III (green), and IV (brown) are colored differently in this figure for ease of recognition. α-Helics and β-sheets are labeled according to their NMR structures. (B). The structure shown in this panel is the same structure as shown in panel A, but with a 90° horizontal rotation. (TIF) [file pone.0027185.s003.tif]
